# Robust kernel extreme learning machines for postgraduate learning performance prediction

**DOI:** 10.1016/j.heliyon.2024.e40919

**Published:** 2024-12-09

**Authors:** Hongxing Gao, Tianzi Xu, Nan Zhang

**Affiliations:** a Faculty of Education, Shaanxi Normal University, Xi'an, 710062, China; b Graduate School, Wenzhou University, Wenzhou, 325035, China; c Faculty of Education, Wenzhou University, Wenzhou, 325035, China; d College of Computer Science and Artificial Intelligence, Wenzhou University, Wenzhou, 325035, China

**Keywords:** Extreme learning machine, Robust learning, Postgraduate learning performance prediction

## Abstract

In the context of graduate learning in China, mentors are the teachers with the highest frequency of contact and the closest relationships with postgraduate students. Nevertheless, a number of issues pertaining to the relationship between mentors and postgraduate students have emerged with increasing frequency in recent years, resulting in a notable decline in the quality of graduate education. In this paper, we investigate the influence of the relationship between mentors and postgraduate students on the postgraduate learning performance, with postgraduate students' admission motivation and learning pressure acting as moderating variables. In practice, outliers often appear during the data collection stage, and they have a significant impact on the convergence speed and prediction accuracy of machine learning models. In order to mitigate the impact of outliers, we propose a novel kernel extreme learning machine model that is robust to outliers and name it a robust kernel extreme learning machine (RK-ELM). The RK-ELM model can automatically detect any data that may be corrupted by uncertain disturbances, thereby enhancing the robustness and generalization ability of the model. We take 873 full-time postgraduate students from universities in Zhejiang Province, China as the research object, and then form a dataset based on the postgraduates’ questionnaire results and their grade point averages in the current academic year. Experimental results show that: 1) RK-ELM is an effective model for predicting postgraduate learning performance; 2) The relationship between mentors and postgraduates has a significant impact on learning performance, but it cannot directly predict learning performance; 3) The combination of the relationship between mentors and postgraduates and enrollment motivation can be used to predict learning performance, where the former can predict learning performance by influencing learning pressure.

## 1 Introduction

Education, technology, and talent represent the fundamental and strategic support for the comprehensive construction of a modern country [[1], [2], [3]]. Graduate education is the most crucial combination of the first productive force of technology, the first resource of talent, and the first driving force of innovation [4,5]. In other words, it plays an important role in cultivating innovative talents, improving innovation capability, serving economic and social development, and promoting the modernization of the national governance system and governance capability. As the primary providers of graduate education, mentors have the most frequent contact and the closest relationships with postgraduate students, whose educational performance is closely related to the quality of graduate education [6]. The relationship between mentors and postgraduate students (which can be abbreviated as MP-relationship) has a direct impact on mentors' willingness to guide, on postgraduate students' willingness to learn and to interact with them, and thus on the quality of postgraduate education and the effectiveness of mentor team building. A good MP-relationship encourages both parties to constantly gain spiritual exchanges and life insights in the process of knowledge exploration and scientific research exploration. This is not only conducive to the establishment of trust and cooperation between mentors and postgraduate students, but also conducive to the construction of a good academic atmosphere and the inheritance of academic culture. In recent years, with the continuous expansion of the scale of graduate education in China, the types of MP-relationships have become increasingly diverse and complex [7]. Most scholars believe that MP-relationships affect the academic performance of graduate students, and have proposed construction strategies for harmonious MP-relationship. However, the correlation between the MP-relationship and the learning performance of postgraduate students has not been thoroughly investigated. This paper focuses on exploring the MP-relationship and the postgraduate students' learning performance, and how the MP-relationship affects postgraduate students’ learning performance.

Recently, the application of artificial intelligence in the field of education has received widespread attention [8,9]. Johnson et al. [10] believe that artificial intelligence not only brings new opportunities for educational evaluation, but also provides greater opportunities for teaching mode reform, personalized learning, and open dialogue, which is particularly important for the development of future education. The core of artificial intelligence-based educational services is the various models constructed by machine learning algorithms. Machine learning refers to a set of algorithmic program combinations that simulate human learning behavior, with the goal of learning hidden patterns from large amounts of existing data and using them for prediction or classification [[11], [12], [13]]. That is, machine learning can use algorithms to learn patterns from educational data to predict the future behavior and performance of educational research objects, where the model interpretability is the key factor for educational practitioners to trust artificial intelligence services. Gao et al. [14] made use of three machine learning models (i.e., selection decision tree, random forest, and boosting) as candidate models for predicting undergraduate learning quality. Zhu et al. [15] proposed an efficient evaluation model based on an improved extreme learning machine, which can provide a reasonable reference for universities to deepen the reform and innovation of education and further improve the level of international education. Sorour et al. [16] labeled students’ behavior and constructed an interpretable learning performance prediction model using a tree structure algorithm. Zhang et al. [17] constructed a learning performance prediction model using genetic algorithms, which explained important variables required for predicting learning performance and the relationships between these important variables.

Extreme learning machine (ELM) is a typical machine learning model that has gained increasing interest from various research fields [[18], [19], [20]]. By replacing the feature mapping in ELM with kernel functions, ELM with kernel (KELM) is developed to solve classification and regression problems [21,22]. Li et al. [23] developed an educational building evacuation model based on an improved KELM, which can effectively ensure the timeliness and accuracy of risk assessment in educational buildings. Although KELM is more precise and efficient, it still cannot handle the outliers that often occur during the educational data collection stage. To address this issue, we propose a novel robust kernel extreme learning machine (RK-ELM) on the basis of KELM, which improves the robustness and generalization ability of KELM. Unlike existing ELM methods, RK-ELM introduces a new robust learning term that can automatically detect outliers under uncertain disturbances and extract effective feature information. Furthermore, RK-ELM is used to investigate the MP-relationship and the postgraduate students’ learning performance. The main contributions of this paper are as follows:

(1) To address the drawbacks of KELM's inability to handle outliers, this paper proposes a method to automatically detect outliers under uncertain disturbances and calls it as RK-ELM. RKELM can capture the nonlinear structural relationships between data and automatically discover outliers when predicting the relationship between research and postgraduate learning performance.
(2) We construct an educational dataset using the survey results of 873 full-time master's students, which consists of four principal dimensions: MP-relationship, enrollment motivation, learning pressure, and learning attitude. Experimental results show that these four dimensions are all useful in predicting postgraduate learning performance, with MP relationship having a significant impact on the learning performance.

We organize the remainder of the paper as follows. Section 2 introduces the related works, including KELM and MP-relationship. Section 3 details the proposed RK-ELM, including the learning procedure and model analysis. In Section 4, experiment results show the MP-relationship and postgraduate students’ learning performance. Finally, some conclusions are given in Section 6.

## 2 Related works

This section first introduces ELM and KELM, and then presents research on MP-relationship.

### 2.1 ELM and KELM

**Fig. 1:**
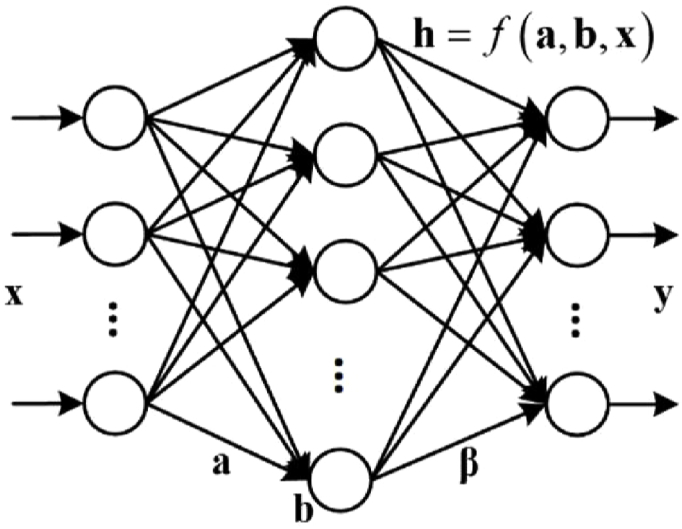
Graphical representation of the structures of ELM. It consists of an input layer **x**, a hidden layer **h**, and an output layer **y**.

ELM is an efficient single hidden layer feedforward neural network [18,24], which consists of an input layer **x**, a hidden layer **h**, and an output layer **y**. After selecting the appropriate number of hidden layer nodes, the whole learning procedure of ELM is completed only once, without the need for iteration. As shown in Fig. 1, after randomly determining the connection weights **a** and the hidden layer biases **b**, the output of the hidden layer **y** can be calculated by:

(1) h=f(a,b,x),

where f(·) denotes the activation function on the hidden layer. When a training dataset $X={\left\{x\right\}}_{n=1}^{N}\in R^{N\times D},Y={\left\{y\right\}}_{n=1}^{N}\in R^{N\times K}$ is given, the output of the hidden layer is calculated by $H={\left\{f\left(a,b,x\right)\right\}}_{n=1}^{N}\in R^{N\times J}$, and the output weights β is calculated using the least squares method:

(2) $$\begin{array}{l} \begin{array}{cc} \min_{\beta } & L_{ELM}=\frac{1}{2}{\left\|\beta \right\|}^{2} \end{array}+\frac{C}{2}{\left\|Y-H\beta \right\|}^{2}\\ \$and\$\\ \min_{\beta }L_{RoK-ELM}=\frac{1}{2}\text{Tr}\left(\beta^{T}K\left(X,X\right)\beta \right)+\frac{C}{2}\sum_{i}w_{i}{\left\|y_{i}-K\left(x_{i},X\right)\beta \right\|}^{2} \end{array}\text{,}$$

where *C* is the regularization parameter. When the derivative of $L_{ELM}$ with β is equal to zero, we obtain the solution of the output weights β:

(3) $$\beta =\left\{ \begin{array}{cc} {\left(I/C+H^{T}H\right)}^{-1}H^{T}Y, & N\geq J\\ H^{T}{\left(I/C+HH^{T}\right)}^{-1}Y, & N<J \end{array}\right.\text{.}$$

When a new instance $x^{\prime }$ appears, the corresponding output is:

(4) $$G\left(x^{\prime }\right)=f\left(a,b,x^{\prime }\right)\beta =f\left(a,b,x^{\prime }\right)H^{T}{\left(I/C+HH^{T}\right)}^{-1}Y\text{.}$$

The traditional ELM model maps input data to high-dimensional features. Moreover, the random setting of input weights a and the hidden layer biases b also leads to the instability of ELM. By introducing the kernel method into ELM, KELM directly obtains the nonlinear transformation of the low-dimensional input data from the kernel function, simplifying the computation and avoiding the random determination of **a** and **b** [21]. In KELM, the mapping from the input layer to the hidden layer is unknown, which replace $f\left(a,b,x^{\prime }\right)H^{T}$ and $HH^{T}$ by a kernel function with:

(5) $$\begin{array}{l} f\left(a,b,x^{\prime }\right)H^{T}≜K\left(x^{\prime },X\right)=\left[\begin{array}{cccc} K\left(x^{\prime },x_{1}\right) & K\left(x^{\prime },x_{2}\right) & \cdots & K\left(x^{\prime },x_{N}\right) \end{array}\right]\text{,}\\ HH^{T}≜K\left(X,X\right)=\left[\begin{array}{cccc} K\left(x_{1},x_{1}\right) & K\left(x_{1},x_{2}\right) & \cdots & K\left(x_{1},x_{N}\right)\\ K\left(x_{2},x_{1}\right) & K\left(x_{2},x_{2}\right) & \cdots & K\left(x_{2},x_{N}\right)\\ \vdots & \vdots & \ddots & \vdots \\ K\left(x_{N},x_{1}\right) & K\left(x_{N},x_{2}\right) & \cdots & K\left(x_{N},x_{N}\right) \end{array}\right]\text{,} \end{array}$$

where $K\left(x^{\prime },x\right)$ denotes the kernel function between two instances $x^{\prime }$ and **x**. In this way, and the output weights $\beta^{\prime }$ is calculated using the least squares method:

(6) $$\min_{\beta^{\prime }}\begin{array}{l} \;\frac{1}{2}\text{Tr}\left({\beta^{\prime }}^{T}K\left(X,X\right)\beta^{\prime }\right)+\frac{C}{2}{\left\|Y-K\left(X,X\right)\beta^{\prime }\right\|}^{2} \end{array}\text{,}$$

and the corresponding output of the new instance $x^{\prime }$ in KELM is:

(7) $$G\left(x^{\prime }\right)=K\left(x^{\prime },X\right){\left(I/C+K\left(X,X\right)\right)}^{-1}Y=K\left(x^{\prime },X\right)\beta^{\prime }\text{.}$$

### 2.2 Research on MP-relationship

In the context of postgraduate learning in China, mentors are the teachers with the highest frequency of contact and the closest relationships with postgraduate students. Chinese scholars' research on the MP-relationship mainly focuses on influencing factors, existing problems, and improvement strategies. Hu [25] believed that the MP-relationship is a special social interpersonal relationship, which is a multi-dimensional relationship system formed by direct communication activities such as knowledge teaching, scientific research, ideological exchange, and behavioral guidance. Ji [26] pointed out that the MP-relationship is a guidance relationship, evaluation relationship, and academic cooperation relationship between mentors and postgraduate students, among which the academic cooperation relationship is derived from the guidance relationship and evaluation relationship. Chen and Hu [27] believed that mentors' task arrangements, communication attitudes, daily interactions, and assistance in understanding graduate students can all affect the development of MP-relationships. Wang et al. [28] pointed out that the control variables, academic status, lifestyle habits, and psychological status of postgraduate students all have a significant impact on MP-relationships, with these four influencing factors having different effects on MP-relationships. Liu [29] pointed out that the root cause of the problem of MP-relationships lies in the insufficient reserve of mentors’ educational literacy and action deviation, insufficient preparation and investment in graduate development conditions, poor two-way interaction between mentors and students, and lack of external guarantee conditions. Xiao [30] proposed that mechanism construction is the foundation for ensuring a harmonious and beautiful MP-relationship: strengthening the training mechanism for teachers and students, improving the mechanism for safeguarding the rights and interests of mentors and postgraduate students, and establishing a positive interactive educational feedback mechanism.

Most international scholars concur that harmonious MP-relationships can be established through the implementation of methodologies such as equal communication, the establishment of an academic atmosphere, and the enhancement of the mentor system. For example, Rose et al. [31] proposed the model of ideal mentors, which suggests that most graduate students prefer mentors with high moral character and strong guidance abilities. Eley and Jennings [32] believed that teachers’ social and emotional competence is pivotal in fostering harmonious MP-relationships. In particular, the presence of mentors with social and emotional abilities can facilitate interaction and engagement between mentors and postgraduate students in the classroom. Additionally, these abilities can contribute to the formation of emotional bonds between postgraduate students and mentors, which are crucial for maintaining positive relationships. Stein et al. [33] and Rogers et al. [34] discovered that postgraduate students who have a strong connection with their mentors tend to perform better than those who are less engaged with their mentors. With the ongoing expansion of graduate education scale and the rise in negative events related to research guidance, the forms of MP-relationships are becoming increasingly diverse and intricate. Nevertheless, the research on the correlation between the MP-relationship and the graduate learning performance remains limited.

## 3 Methodology

This section mainly introduces the proposed RK-ELM model, including the learning procedure and the relationship to related works.

### 3.1 Methodology

This paper explores the correlation between the MP-relationship and the postgraduate learning performance, using enrollment motivation and learning pressure as moderating variables. Outliers often appear in the data collection process, which not only greatly affect the convergence speed of machine learning models, but also has significant side effects on the prediction accuracy of machine learning models. In order to reduce the impact of {outliers}, this paper proposes a robust kernel extreme learning machine (RK-ELM) model based on the KELM model. RK-ELM can detect automatically outliers under uncertain disturbances, thereby improving the robustness and generalization ability of the model. Given the training instances $X={\left\{x_{n}\right\}}_{n=1}^{N}\in R^{N\times D},Y={\left\{y_{n}\right\}}_{n=1}^{N}\in R^{N\times K}$ and kernel function $K\left(x,x^{\prime }\right)$, the objective function of RK-ELM consists of regularization term and robust prediction error term:

(8) $$\min_{\beta^{\prime },\varepsilon }\;\frac{1}{2}\text{Tr}\left({\beta^{\prime }}^{T}K\left(\tilde{X},\tilde{X}\right)\beta^{\prime }\right)+\frac{C}{2}\sum_{n}1_{MSE_{n}\leq \varepsilon }{\left\|K\left(x_{n},\tilde{X}\right)\beta^{\prime }-y_{n}\right\|}_{2}^{2}\text{,}$$

where $\beta^{\prime }$ is the output weights, *C* is the regularization parameter, the parameter ε is determined based on the prediction error of the instances, $\tilde{X}$ is the set of instances in $\tilde{X}$ that remove outliers, $MSE_{n}={\left\|K\left(x_{n},\tilde{X}\right)\beta^{\prime }-y_{n}\right\|}_{2}^{2}$ denotes the prediction error of the *n*-th instance, and assumes that *r*% of the instances are outliers. If $MSE_{n}\geq \varepsilon$, then $1_{MSE_{n}\leq \varepsilon }=0$ and the *n*-th instance is an outlier, otherwise $1_{MSE_{n}\leq \varepsilon }=1$ and the *n*-th instance is a normal instance. By observing Eq. (8), we can find the following points: 1) Compared to KELM, the main innovation of the proposed RK-ELM model is the addition of an automatic anomaly detection mechanism, which helps to improve the performance of the proposed model and can be applied to other improved KELM models; 2) The optimization of RK-ELM is a joint optimization problem of the output weights $\beta^{\prime }$ and the parameter ε, where the parameter ε can be automatically determined based on the outlier rate *r*% and the detailed optimization is given in the next subsection.

### 3.2 Optimization and learning

**Table:** 

**Algorithm 1.** Learning procedure of RK-ELM
**Input:** The training instances $X={\left\{x\right\}}_{n=1}^{N}\in R^{N\times D},Y={\left\{y\right\}}_{n=1}^{N}\in R^{N\times K}$, the kernel function $K\left(x,x^{\prime }\right)$, the outlier rate *r*%, the maximum number of iterations $i_{\max}$.
**Output:** The output weights $\beta^{\prime }$ and the “clean” instances $\tilde{X}$ that removes outliers.
1: **Initialize**$\tilde{X}=X$.
2: **for**$i=1:i_{\max}$**do**
3: Update the output weights $\beta^{\prime }$ using Eq. (11).
4: Calculate the mean square error of each instance $MSE_{n}={\left\|K\left(x_{n},\tilde{X}\right)\beta^{\prime }-y_{n}\right\|}_{2}^{2}$.
5: Sort ${\left\{MSE_{n}\right\}}_{n=1}^{N}$ from largest to smallest and obtain the mean square error ${\left\{MS{E^{\prime }}_{n}\right\}}_{n=1}^{N}$ after sorting.
6: Calculate the parameter $\varepsilon =MSE_{N\times r\%}^{\prime }$, and obtain the “clean” instances $\tilde{X}$ that removes outliers.
7: **end for**
8: **return** the output weights $\beta^{\prime }$ and the “clean” instances $\tilde{X}$.

The optimization of RK-ELM can be achieved by iteratively learning the output weights $\beta^{\prime }$ and the parameter ε: (1) Fixed the parameter ε and learn the output weights $\beta^{\prime }$; (2) Fixed the output weights $\beta^{\prime }$ and learn the parameter ε. As shown in Algorithm 1, if the “clean” instances $\tilde{X}$ remains unchanged or the maximum number of iterations is reached (i.e., the maximum number of iterations is set to 5 in this paper), then RK-ELM obtains the final model parameters.

#### 3.2.1 Fixed the parameter ε and learn output weights $\beta^{\prime }$

The objective function of RK-ELM with the fixed parameter ε degenerates into:

(9) $$\begin{array}{l} \min_{\beta^{\prime }}f\left(\beta^{\prime }\right)=\frac{C}{2}\sum_{n}1_{MSE_{n}\leq \varepsilon }{\left\|K\left(x_{n},\tilde{X}\right)\beta^{\prime }-y_{n}\right\|}_{2}^{2}+\frac{1}{2}\text{Tr}\left({\beta^{\prime }}^{T}K\left(\tilde{X},\tilde{X}\right)\beta^{\prime }\right)=\frac{C}{2}{\left\|K\left(\tilde{X},\tilde{X}\right)\beta^{\prime }-\tilde{Y}\right\|}_{2}^{2}+\frac{1}{2}\text{Tr}\left({\beta^{\prime }}^{T}K\left(\tilde{X},\tilde{X}\right)\beta^{\prime }\right)\; \end{array}\text{,}$$

where $\tilde{Y}$ is the label corresponding to the “clean” instances $\tilde{X}$ that removes outliers. If the partial derivative of $f\left(\beta^{\prime }\right)$ is equal to zeros, we have:

(10) $$\frac{\partial f\left(\beta^{\prime }\right)}{\partial \beta^{\prime }}=C\cdot K\left(\tilde{X},\tilde{X}\right)\left(K\left(\tilde{X},\tilde{X}\right)\beta^{\prime }-\tilde{Y}\right)+K\left(\tilde{X},\tilde{X}\right)\beta^{\prime }=0\text{.}$$

In this way, the solution of the output weights $\beta^{\prime }$ is:

(11) $$g\left(x^{\prime }\right)=K\left(x^{\prime },\tilde{X}\right){\left(\frac{I}{C}+K\left(\tilde{X},\tilde{X}\right)\right)}^{-1}\tilde{Y}\text{.}$$

#### 3.2.2 Fixed learn output weights $\beta^{\prime }$ and the parameter ε

Fixed output weights $\beta^{\prime }$, the mean square error of each instance is obtained by $MSE_{n}={\left\|K\left(x_{n},\tilde{X}\right)\beta^{\prime }-y_{n}\right\|}_{2}^{2}$. The parameter ε can be obtained based on the outlier rate *r*% and ${\left\{MSE_{n}\right\}}_{n=1}^{N}$. Specifically, sorting ${\left\{MSE_{n}\right\}}_{n=1}^{N}$ from largest to smallest, we obtain ${\left\{MS{E^{\prime }}_{n}\right\}}_{n=1}^{N}$. Then, the parameter ε is equal to $MSE_{N\times r\%}^{\prime }$, and the “clean” instances $\tilde{X}$ that removes outliers is obtained at the same time.

After the training is completed, the corresponding output of the new instance $x^{\prime }$ in RK-ELM is:

(12) $$g\left(x^{\prime }\right)=K\left(x^{\prime },\tilde{X}\right){\left(\frac{I}{C}+K\left(\tilde{X},\tilde{X}\right)\right)}^{-1}\tilde{Y}\text{.}$$

### 3.3 Optimization and learning

#### 3.3.1 Relationship to related works

In the ELM algorithm, the activation function on the hidden layer is initially employed to derive the nonlinear mapping of the sample. Subsequently, the output weight is calculated by solving the least squares problem. Many scholars have sought to enhance the capabilities of traditional extreme learning machines, to improve their performance in classification and prediction tasks. For example, Wang et al. [35] demonstrated that when the activation function is a radial basis function, there exists an input weight value such that the output of the hidden layer of the instance is full column rank or full row rank. On this basis, Chen et al. [36] extended the model to ELM, where the activation function is the sigmoid function. Incremental ELM (IELM) [37] enables the selection of suitable nodes from the candidate nodes and their incorporation into the hidden layer during the training process, thereby contributing to better algorithm performance. Bidirectional ELM (BELM) [38] reduces the network size on the basis of IELM. Pruning ELM (PELM) [39,40] removes hidden nodes that contribute less to training performance, thereby achieving the goal of reducing network size in a manner similar to BELM while ensuring algorithm performance. However, these models ignore the impact of outliers on ELM. The RK-ELM model proposed in this paper can effectively identify outliers and reduce the impact of outliers on the model. Furthermore, in comparison to the aforementioned models, the RK-ELM model exhibits the following characteristics: 1) RK-ELM employs the kernel method to substitute for the hidden layer outputs of the aforementioned models, thus avoiding random determination of input weights and reducing the randomness and uncertainty of the model; 2) Although the RK-ELM model necessitates iterative computation, it is capable of detecting outliers in a reduced number of steps, thereby improving the computational efficiency of the RK-ELM model. Recently, some robust ELM methods have been proposed to deal with noise problems, such as generalized outlier robust ELM (GOR-ELM) [41,42] and robust KELM (RoK-ELM) [43]. Specifically, GOR-ELM makes use of the $l_{21}$ norm to constraint the prediction error, and its optimization problem can be rewritten as:

(13) $$\begin{array}{l} \min_{\beta ,Z}\;L_{GOR-ELM}=\frac{C}{2}{\left\|Y-H\beta \right\|}_{2,1}+\frac{\lambda }{2}\left(1-\alpha \right){\left\|\beta \right\|}_{F}^{2}+\frac{\lambda \alpha }{2}{\left\|Z\right\|}_{2,1}\\ \;s.t.\;\beta -Z=0 \end{array}\text{,}$$

where C,λ,α are the regularization parameters, ${\left\|\cdot \right\|}_{F}$ and ${\left\|\cdot \right\|}_{2,1}$ are the Frobenius norm and the

$l_{21}$ norm, respectively. RoK-ELM provides better reliability of the instances by unevenly weighting each instance, and its optimization problem is rewritten as:

(14) $$\min_{\beta }L_{RoK-ELM}=\frac{1}{2}\text{Tr}\left(\beta^{T}K\left(X,X\right)\beta \right)+\frac{C}{2}\sum_{i}w_{i}{\left\|y_{i}-K\left(x_{i},X\right)\beta \right\|}^{2}\text{,}$$

where *C* is the regularization parameter, and $W=diag\left[w_{1},w_{2},\cdots ,w_{N}\right]$ is the weighting matrix for the prediction error. It can be observed that GOR-ELM incorporates the $l_{21}$ norm into the optimization objective, thereby achieving denoising capability and enhanced robustness. In contrast, RK-ELM and RoK-ELM provide better reliability of the instances through instance selection or instance weighting. Furthermore, RoK-ELM introduces a weight matrix of instances to mitigate the impact of outliers, while RK-ELM introduces a novel robust learning term that can automatically detect outliers under uncertain disturbances. In other words, RK-ELM can learn the “clean” instances without outliers, where the detection of outliers helps to improve the learning performance of the proposed model.

#### 3.3.2 Critical analysis

The proposed RK-ELM model is an efficient machine learning algorithm based on kernel methods, which can be applied in fields such as pattern recognition, classification, and regression. The RK-ELM introduces a novel robust learning term that is capable of automatically detecting outliers in the presence of uncertain disturbances and extracting effective feature information. However, if there are no outliers in the collected instances, the proposed RK-ELM degenerates into KELM. In other words, if the quality of the training instances is satisfactory, the performance of RK-ELM and RK-ELM is identical. Furthermore, it can be observed that the objective function of RK-ELM contains a regularization hyperparameter, denoted by *C*, which influences the performance of RK-ELM. In summary, the RK-ELM algorithm is suitable for classification or regression problems involving outliers.

## 4 Experiment and analysis

### 4.1 Data collection and experimental setting

We conducted a questionnaire survey on 873 full-time master's degree students in universities in Zhejiang Province via an online survey platform. A total of 818 valid questionnaires were returned, representing an effective participation rate of 93.70 %. The survey respondents consisted of 368 male students (42.15 %) and 505 female students (57.85 %), 374 academic masters (42.84 %) and 499 professional masters (57.16 %), 430 liberal arts postgraduates (49.26 %) and 443 science and technology postgraduates (50.74 %), and 337 urban students (38.60 %) and 536 rural students (61.40 %). In addition to the personal information about postgraduate students, the questionnaire consists of four principal dimensions: enrollment motivation, learning pressure, MP-relationship, and learning attitude. The details are as follows:

(1) **Enrollment motivation**: The enrollment motivation is based on the graduate entrance motivation survey questionnaire, which is compiled by Wang et al. [44] and comprises a total of eight questions, including three components (i.e., employment orientation, professional orientation, and blind orientation).
(2) **Learning pressure**: The learning pressure is based on the postgraduate learning pressure form developed by Liu (2015), which consists of a total of 19 questions, including four components (i.e., personal pressure, social pressure, family pressure, and school pressure).
(3) **MP-relationship**: The MP-relationship is primarily concerned with the mentor-postgraduate relationship and the growth of postgraduates questionnaire compiled by Li [45], with a total of 34 questions, including four components (i.e., basic information about the mentor, reasons for choosing the mentor, the academic support of the mentor, and the academic cooperation of the mentor and postgraduates).
(4) **Learning attitude**: The learning attitude mainly refers to the self-assessment form of academic achievement compiled by Wang [46], with a total of 14 questions, mainly including three components (i.e., learning initiative, efficacy and execution).

In this way, we form a dataset (i.e., {**X=[X0;X1;X2;X3;X4],Y**}) based on postgraduates’ questionnaire results and grade point average (GPA) in the current academic year, where **X0** denotes the personal information about postgraduate students, **X1** denotes the enrollment motivation, **X2** denotes the learning pressure, **X3** denotes the MP-relationship, **X4** denotes the learning attitude, **Y** denotes the GPA in the current academic year. For this dataset, we normalize the attributes by normalizing the value range of each attribute to between 0 and 1. We divide the dataset into five parts (i.e., three of which are used for training, one for parameter selection, and one for testing) and use the cross-validation method to validate the proposed model. The parameter settings of the RK-ELM model are shown in Table 1, where the kernel function selects the Gaussian kernel function, the regularization parameter C is selected from {2^−20^, 2^−19^, …, 2^19^, 2^20^}, and the outlier rate r% is selected from {5 %, 10 %, 15 %, 20 %}, and the maximum number of iterations is 5.

**Table 1:** Parameter settings of the RK-ELM model.

Parameters	Settings
Kernel function	the Gaussian kernel function
Regularization parameter *C*	{2^−20^, 2^−19^, …·, 2^19^, 2^20^}
Outlier rate *r*%	5 %, 10 %, 15 %, 20 %
The maximum number of iterations	5

### 4.2 Results and analysis

**Fig. 2:**
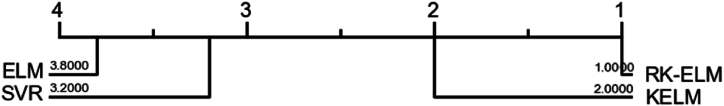
Comparison of RK-ELM against contrast methods with the Wilcoxon-Holm test.

We first verify the validity of RK-ELM on different data combinations. Table 2 shows the experimental results of RK-ELM and contrast methods (i.e., ELM, Support Vector Regression (SVR), and KELM) in terms of the root mean square error, where the best results highlighted and the outlier rate in RK-ELM is set to 5 %. The smaller the value of the root mean square error, the better the performance of the algorithm. By observing Table 2, we can find the following points: 1) Compared to ELM and SVR, KELM has performed well but is still affected by outliers. For instance, the average prediction error of KELM on [X0, X1, X2] is 0.0006, the standard deviation is 0.0013 and is greater than the mean prediction error; 2) Although RK-ELM is significantly better than contrast methods in terms of “Friedman Ranking”, the Wilcoxon-Holm test is conducted to provide further insight into the performance of RK-ELM. Fig. 2 presents a comprehensive comparison of RK-ELM against contrast methods (i.e., ELM, SVR, and KELM) with the Wilcoxon-Holm test [47]. The Wilcoxon-Holm score of RK-ELM is notably lower than that of contrast methods, indicating that it is capable of automatically detecting outliers under uncertain disturbances and extracting effective feature information.

**Table 2:** Prediction results of RK-ELM and contrast methods under different data combinations in terms of the root mean square error. The best results are highlighted.

Data Combination	ELM	SVR	KELM	RK-ELM
**X0, X1**	0.1446 ± 0.0022	0.1549 ± 0.0037	0.0145 ± 0.0069	**0.1826e-03 ± 0.0388e-03**
**X0**, **X2**	0.1435 ± 0.0028	0.1443 ± 0.0097	0.0220 ± 0.0035	**0.1075e-04 ± 0.0240e-04**
**X0**, **X3**	0.1424 ± 0.0047	0.1098 ± 0.1098	0.5015e-05 ± 0.0792e-05	**0.2800e-05 ± 0.0130e-05**
**X0**, **X4**	0.1430 ± 0.0042	0.1507 ± 0.0047	0.0368 ± 0.0055	**0.0042 ± 0.0011**
**X0**, **X1**, **X2**	0.1421 ± 0.0023	0.1127 ± 0.0017	0.0006 ± 0.0013	**0.2795e-05 ± 0.0373e-05**
**X0**, **X1**, **X3**	0.1413 ± 0.0033	0.0657 ± 0.0137	0.2491e-05 ± 0.0202e-05	**0.1748e-05 ± 0.0074e-05**
**X0**, **X1**, **X4**	0.1420 ± 0.0035	0.1389 ± 0.0034	0.1188e-04 ± 0.0310e-04	**0.5638e-05 ± 0.0507e-05**
**X0**, **X2**, **X3**	0.1404 ± 0.0041	0.0380 ± 0.0091	0.1769e-05 ± 0.0073e-05	**0.1266e-05 ± 0.0089e-05**
**X0**, **X2**, **X4**	0.1408 ± 0.0039	0.1099 ± 0.0038	0.0070 ± 0.0039	**0.2708e-05 ± 0.0340e-05**
**X0**, **X3**, **X4**	0.1395 ± 0.0051	0.0609 ± 0.0121	0.2564e-05 ± 0.0188e-05	**0.1637e-05 ± 0.0055e-05**
**X0**, **X1**, **X2**, **X3**	0.1390 ± 0.0028	0.0173 ± 0.0131	0.1402e-05 ± 0.0077e-05	**0.1049e-05 ± 0.0069e-05**
**X0**, **X1**, **X2**, **X4**	0.1394 ± 0.0037	0.0730 ± 0.0089	0.3365e-05 ± 0.1257e-05	**0.1619e-05 ± 0.0177e-05**
**X0**, **X1**, **X3**, **X4**	0.1387 ± 0.0042	0.0399 ± 0.0165	0.1723e-05 ± 0.0104e-05	**0.1173e-05 ± 0.0105e-05**
**X0**, **X2**, **X3**, **X4**	0.1377 ± 0.0043	0.0117 ± 0.0086	0.1474e-05 ± 0.0107e-05	**0.1019e-05 ± 0.0049e-05**
**X0**, **X1**, **X2**, **X3**, **X4**	0.1364 ± 0.0036	0.0025 ± 0.0050	0.1223e-05 ± 0.0072e-05	**0.8774e-06 ± 0.0537e-06**
**Friedman Ranking**↓	3.80 ± 0.41	3.20 ± 0.41	2.00 ± 0.00	**1.00 ± 0.00**

Furthermore, Table 3 shows the root mean square error of RK-ELM's prediction results under different data combinations, where the best results highlighted. From Table 3, we have the following observations: 1) When the four dimensions are predicted separately, RK-ELM trained based on MP-relationship (i.e., [X0; X3]) performs the best, while RK-ELM trained based on learning attitude (i.e., [X0; X4]) performs the worst. This indicates that MP-relationship has a significant impact on learning performance; 2) When combining any two dimensions for prediction, RK-ELM trained based on the combination of learning pressure and MP-relationship (i.e., [X0; X2; X3]) performs best. Furthermore, RK-ELM trained based on the combination with MP-relationship performs better than the combination without MP-relationship; 3) When combining any three dimensions for prediction, RK-ELM trained based on the combination of learning pressure, MP-relationship, and learning attitude (i.e., [X0; X2; X3; X4]) achieves better prediction results in most cases, while RK-ELM trained based on the enrollment motivation, learning pressure, and MP-relationship (i.e., [X0; X1; X2; X3]) achieves the best prediction result when r% is equal to 15 %. This indicates that learning pressure and MP-relationship are more important when combining any three dimensions for prediction; 4) RK-ELM trained based on the combination of four dimensions yields the best results, indicating that all four dimensions are useful in postgraduate learning performance prediction.

**Table 3:** RK-ELM's prediction results under different data combinations in terms of the root mean square error.

Data Combination	The Proposed RK-ELM Model			
	*r*% = 5 %	*r*% = 10 %	*r*% = 15 %	*r*% = 20 %
**X0, X1**	0.1826e-03	0.9597e-04	0.6736e-04	0.5599e-04
**X0**, **X2**	0.1075e-04	0.6253e-05	0.5295e-05	0.4472e-05
**X0**, **X3**	**0.2800e-05**	**0.2275e-05**	**0.2023e-05**	**0.1853e-05**
**X0**, **X4**	0.0042	0.7373e-04	0.3304e-04	0.2132e-04
**X0**, **X1**, **X2**	0.2795e-05	0.2218e-05	0.1916e-05	0.1695e-05
**X0**, **X1**, **X3**	0.1748e-05	0.1527e-05	0.1290e-05	0.1154e-05
**X0**, **X1**, **X4**	0.5638e-05	0.4555e-05	0.4055e-05	0.3372e-05
**X0**, **X2**, **X3**	**0.1266e-05**	**0.1128e-05**	**0.9643e-06**	**0.8610e-06**
**X0**, **X2**, **X4**	0.2708e-05	0.2251e-05	0.1810e-05	0.1675e-05
**X0**, **X3**, **X4**	0.1637e-05	0.1342e-05	0.1194e-05	0.1066e-05
**X0**, **X1**, **X2**, **X3**	**0.1049**e-**05**	**0.9169e-06**	**0.7768e-06**	0.7643e-06
**X0**, **X1**, **X2**, **X4**	0.1619e-05	0.1340e-05	0.1208e-05	0.1098e-05
**X0**, **X1**, **X3**, **X4**	0.1173e-05	0.1028e-05	0.9072e-06	0.8169e-06
**X0**, **X2**, **X3**, **X4**	0.1019e-05	0.9111e-06	0.7852e-06	**0.7102e-06**
**X0**, **X1**, **X2**, **X3**, **X4**	**0.8774e-06**	**0.7840e-06**	**0.7086e-06**	**0.6550e-06**

**Fig. 3:**
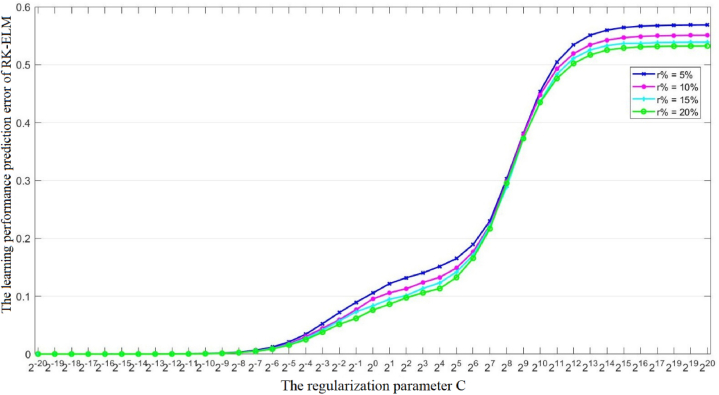
The variations in the learning performance prediction error of RK-ELM with respect to the regularization parameter C on the MP-relationship.

**Fig. 4:**
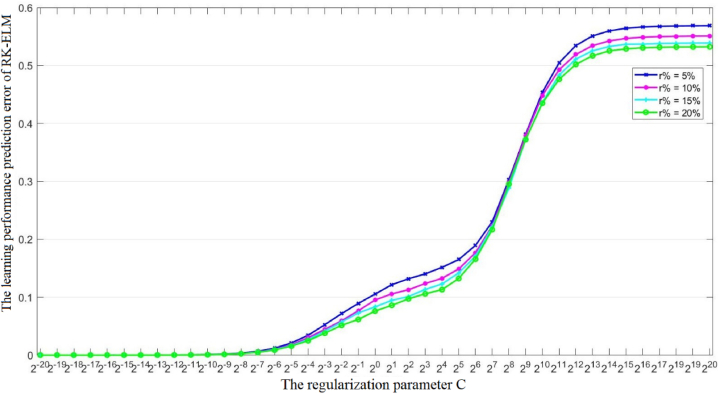
The variations in the learning performance prediction error of RK-ELM with respect to the regularization parameter C on the combination of learning pressure and MP-relationship.

From Table 3, we can also see that the learning performance prediction error of RK-ELM decreases with the increase of the outlier rate r%, indicating that the detection of outliers helps to improve the learning performance of RK-ELM. In addition to the outlier rate *r*%, the regularization parameter *C* also need to be determined beforehand. In other words, the regularization parameter *C* affects the learning performance prediction error of the proposed RK-ELM model. Fig. 3, Fig. 4 show the variations in the learning performance prediction error of RK-ELM with respect to the regularization parameter C on the MP-relationship (i.e., [X0; X3]) and the combination of learning pressure and MP-relationship (i.e. [X0; X2; X3]), respectively. From Fig. 3, Fig. 4, we have the following observations: 1), The trends of the performance change curve of RK-ELM are consistent under different combinations; 2) RK-ELM can achieve good prediction performance when the regularization parameter *C* is less than 2^−10^, which indicates that RK-ELM is robust to parameter *C* to a certain extent; 3) RK-ELM achieves relatively good performance in high outlier rate *r*%, indicating that there is a certain amount of outliers in the dataset and high outlier rate *r*% is beneficial for learning to obtain robust instances $\tilde{X}$.

## 5 Discussion on MP-relationship and postgraduates’ learning performance

### 5.1 MP-relationship has a significant impact on postgraduate students’ learning performance

**Fig. 5:**
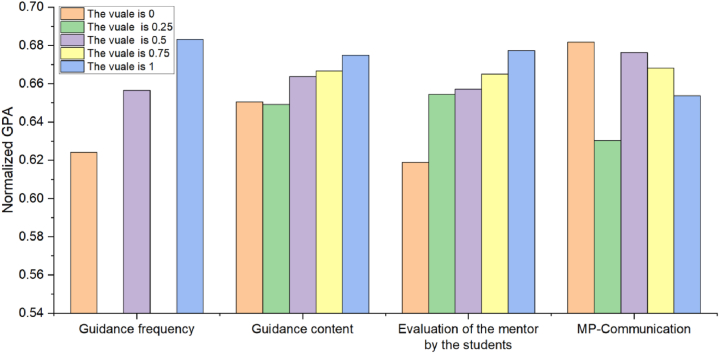
The relationship diagram between MP-relationship and the normalized GPA, where MP-relationship consists of guidance frequency, guidance content, evaluation of the mentor by the students, and MP-Communication.

**Fig. 6:**
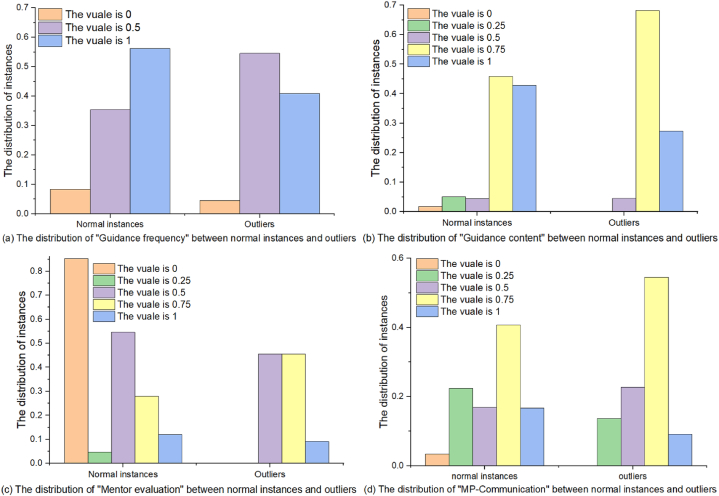
The distribution of MP-relationship between normal instances and outliers, including the distribution of four factors (i.e., guidance frequency, guidance content, evaluation of the mentor by the students (abbr. as “mentor evaluation” in the figure), and MP-Communication).

From Table 2, we have the following observations on the MP-relationship: 1) When the four dimensions are predicted separately, RK-ELM trained based on MP-relationship performs the best; 2) When combining different dimensions for prediction, RK-ELM trained based on the combination with MP-relationship achieves relatively good performance. These observations all indicate that MP-relationship has a significant impact on the postgraduate students' learning performance, which is consistent with the research results of Pan and Gu [48]. Moreover, in order to explore how MP-relationship affects postgraduate students' learning performance, Fig. 5 shows the relationship diagram between MP-relationship and the normalized GPA, where MP-relationship consists of guidance frequency (e.g., how many academic exchange activities are held every month), guidance content (e.g., my mentor assigned me learning tasks based on my interests), evaluation of the mentor by the students (e.g., my mentor is very satisfied with my academic performance), and MP-Communication (e.g., my mentor and I have extensive daily communication activities). We can see that although MP-relationship can affect postgraduate students' GPA to some extent, it is not necessarily that the more harmonious MP-relationship, the higher postgraduate students' GPA. This indicates that the postgraduate students' learning performance prediction requires the participation of other dimensions such as postgraduate students' enrollment motivation and learning attitude in addition to MP-relationship. Furthermore, Fig. 6 reports the distribution of MP-relationship between normal instances and outliers, including the distribution of four factors (i.e., MP-relationship consists of guidance frequency, guidance content, evaluation of the mentor by the students, and MP-Communication). It can be seen that most of outliers detected by RK-ELM appear in situations where MP-relationship is relatively harmonious, which indicates that RK-ELM trained based on MP-relationship (i.e., [X0; X3]) can accurately predict the postgraduates’ learning performance with poor MP-relationship.

### 5.2 The combination of enrollment motivation and MP-relationship can effectively predict postgraduates’ learning performance

Enrollment motivation is an internal driving dimension closely related to graduate learning, which has functions such as guiding direction, eliminating interference, and promoting proactive learning. From Table 3, we can find that RK-ELM trained based on the combination of enrollment motivation and MP-relationship (i.e., [X0; X1; X3]) achieves a prediction error of 1.40e-06 at the 5 % outlier rate, 1.23e-06 at the 10 % outlier rate, 1.034e-06 at the 15 % outlier rate, and 9.30e-07 at the 20 % outlier rate. This indicates that the combination of enrollment motivation and MP-relationship can effectively predict postgraduates' learning performance. Furthermore, enrollment motivation can be divided into different types (i.e., intrinsic enrollment motivation and extrinsic enrollment motivation), where different types of enrollment motivations have different impacts on postgraduates' learning performance. Porter [49] believed that students enrolled with intrinsic motivation can maintain long-term learning satisfaction, while Stefan [50] believed that the learning satisfaction of students enrolled with extrinsic motivation will decrease over time. Table 4 gives the root mean square error of RK-ELM's prediction results under different types of enrollment motivations, where X1-intri represents the intrinsic enrollment motivation and X1-extri represents the extrinsic enrollment motivation. It can be seen that the prediction error of RK-ELM trained based on extrinsic motivation is to the e−02 power, while the prediction error of RK-ELM trained based on intrinsic motivation is to the e−06 power. This indicates that intrinsic motivation is more accurate in postgraduates' learning performance prediction compared to extrinsic motivation. In addition, the prediction error of RK-ELM trained based on both intrinsic and extrinsic motivation is lower than that based on intrinsic motivation, which further indicates that postgraduates who enroll with extrinsic motivation may have an impact on their learning attitude due to unclear learning goals, thereby affecting their learning performance prediction.

**Table 4:** RK-ELM's prediction results under different types of enrollment motivations.

Data Combination	The Proposed RK-ELM Model			
	*r*% = 5 %	*r*% = 10 %	*r*% = 15 %	*r*% = 20 %
**X0, X1**	0.1464e-03	0.7638e-04	0.5377e-04	0.4451e-04
**X0,X1-intri**	**0.9895e-06**	**0.8195e-06**	**0.7684e-06**	**0.7084e-06**
**X0,X1-extri**	0.0371	0.0240	0.0128	0.0071

### 5.3 The combination of MP-relationship and learning pressure can effectively predict postgraduates’ learning performance

For postgraduate students, learning is the primary task, and learning pressure is a norm and an integral part of their learning life. From Table 3, we have the following observations on the learning pressure: 1) When combining any two dimensions for prediction, RK-ELM trained based on the combination of learning pressure and MP-relationship (i.e., [X0; X2; X3]) performs best; 2) When combining any three dimensions for prediction, RK-ELM trained based on the combination with [X0; X2; X3] achieves better prediction results than that without [X0; X2; X3]. These results indicate that the combination of learning pressure and MP-relationship has a significant impact on postgraduate students' learning performance. Academic learning pressure has a dual effect, both as a driving force for learning and as a possible resistance to learning, which mainly depends on the attitude and methods of postgraduate students in dealing with learning pressure. We will further investigate four sources of learning pressure (i.e., personal pressure, social pressure, family pressure, and school pressure). Table 5 gives the root mean square error of RK-ELM's prediction results under different types of learning pressure, where X0,X2-per represents personal pressure, X0,X2-soc represents social pressure, X0,X2-fam represents family pressure, and X0,X2-sch represents school pressure. It can be seen that the prediction error of RK-ELM trained based on X0,X2-per is to the e−02 power, while the prediction error of RK-ELM trained based on other types of learning pressure (i.e., X0,X2-soc, X0,X2-fam, or X0,X2-sch) is to the e−03 power. This indicates that X0,X2-soc, X0,X2-fam, and X0,X2-sch are more accurate in postgraduates' learning performance prediction compared to X0,X2-per. When postgraduate students encounter learning pressure, timely psychological support and assistance from mentors can increase their confidence and courage to overcome academic learning pressure. The correlation coefficient between the harmonious relationship reflected by mentor support and the good academic performance reflected by active response is the highest, which indicates that mentor support directly affects postgraduates' response to learning stress, thereby affecting postgraduates' learning performance.

**Table 5:** RK-ELM's prediction results under different types of learning pressure.

Data Combination	The Proposed RK-ELM Model			
	*r*% = 5 %	*r*% = 10 %	*r*% = 15 %	*r*% = 20 %
**X0, X2**	**0.1075e-04**	**0.6253e-05**	**0.5295e-05**	**0.4472e-05**
**X0, X2-per**	0.0371	0.0205	0.0106	0.0031
**X0, X2-soc**	0.0133	0.0035	0.9752e-03	0.4769e-03
**X0, X2-fam**	0.0165	0.0041	0.0012	0.5795e-03
**X0, X2-sch**	0.0155	0.0053	0.8456e-03	0.3307e-03

## 6 Conclusion

In this paper, we build a novel RK-ELM model that is able to automatically detect outliers under uncertain disturbances, and applies the proposed RK-ELM model to capture the nonlinear structural relationships between MP-relationship and postgraduates' learning performance. RKELM can capture the nonlinear structural relationships between data and automatically discover outliers, where the detection of outliers helps to improve the learning performance of the proposed model. We have formed a survey questionnaire (i.e., it contains MP-relationship, enrollment motivation, learning pressure, and learning attitude) and constructed an educational dataset using the survey results of 873 full-time master's students from universities in Zhejiang Province, China. Experimental results on the educational dataset show that RK-ELM is an effective model for predicting postgraduate learning performance. The relationship between mentors and postgraduates has a significant impact on learning performance, but it cannot directly predict learning performance. Furthermore, the combination of MP-relationship and enrollment motivation can predict learning performance, with the former influencing learning pressure and thus predicting learning performance.

## CRediT authorship contribution statement

**Hongxing Gao:** Writing – original draft, Software, Methodology, Funding acquisition, Data curation. **Tianzi Xu:** Writing – review & editing, Validation, Supervision, Conceptualization. **Nan Zhang:** Methodology, Investigation, Formal analysis.

## Data availability statement

All data analyzed in the current study are available from the first author upon reasonable request. The dataset used in the current study is available at https://pan.baidu.com/s/1W6SLEzimtx6-OkNeDFvkrA?pwd=jayz.

## Funding

This work was supported by the annual research project of Wenzhou 10.13039/501100011788Philosophy and Social Science Planning in 2024 (No.24WSK144YBM), the 10.13039/501100004731Zhejiang Provincial Natural Science Foundation of China (No. LY23F020002), the 2024 Teaching Reform Project of Wenzhou University (“Construction and Practice of Blended Teaching Quality Evaluation System in Colleges and Universities under Learning Input Perspective”), and the 2021 Scientific Research Project of the Education of Zhejiang Province (No. Y202146630).

## Declaration of competing interest

The authors declare that they have no known competing financial interests or personal relationships that could have appeared to influence the work reported in this paper.
